# Draft Genome Sequence of Strain LSUCC0112, a Gulf of Mexico Representative of the Oligotrophic Marine *Gammaproteobacteria*

**DOI:** 10.1128/MRA.00521-19

**Published:** 2019-07-03

**Authors:** Jordan T. Coelho, Michael W. Henson, J. Cameron Thrash

**Affiliations:** a Department of Biological Sciences, University of Southern California, Los Angeles, California, USA; University of Delaware

## Abstract

Here, we present the draft genome sequence of strain LSUCC0112, a cultured representative from the Gulf of Mexico that is phylogenetically close to the OM182 clade within oligotrophic marine *Gammaproteobacteria*. LSUCC0112 shows the potential for aerobic heterotrophy, glycogen synthesis, flagellar motility, and assimilatory sulfate reduction.

## ANNOUNCEMENT

The oligotrophic marine *Gammaproteobacteria* (OMG) consist of five discrete clades with cultured members isolated from the marine environment with oligotrophic lifestyles (1). The OM182 clade within the OMG is putatively ranked near the order *Cellvibrionales*, a group that hosts many abundant marine oligotrophs with global distribution (2). Strain LSUCC0112 was isolated from surface water in Freshwater City, LA, using high-throughput dilution-to-extinction culturing methods (3). Phylogenetic inference with the 16S rRNA gene placed LSUCC0112 as an early diverging member of the OM182 clade of *Gammaproteobacteria* (3). BLASTN versus the NCBI nucleotide database (default settings) using the LSUCC0112 16S rRNA gene sequence (GenBank accession number KU382372) identified Pseudohongiella spirulinae KCTC 32221 (4) (GenBank accession number CP013189) as the closest cultured representative, sharing 96.3% 16S rRNA gene sequence identity with LSUCC0112. Since LSUCC0112 is the only cultured representative in close phylogenetic proximity to the OM182 clade from coastal Gulf of Mexico waters, we sequenced the genome to contribute to the resolution of *Gammaproteobacteria* and *Cellvibrionales* phylogeny as well as the ecophysiology of the OMG.

Cells for genome sequencing were grown in JW4 medium (3) at 25°C and harvested in late exponential phase. Genomic extraction was performed as previously published (5). Both library preparation and Illumina MiSeq sequencing were performed at Argonne National Laboratories. Genomic DNA was sheared via sonication (Covaris, Woburn, MA), and libraries were generated on the Apollo324 system using the PrepX ILMN library kit (TaKaRa, Mountain View, CA), following the manufacturer’s instructions. Resulting libraries were then size selected using BluePippin (Sage Science, Beverly, MA). Sequencing yielded 335,980 raw 2 × 251-bp paired-end reads, which were screened and assembled using A5-miseq (6) using default settings. We assessed the final assembly for completeness and contamination using CheckM using default settings (7). The assembled genome was submitted to the National Center for Biotechnology Information (NCBI) for annotation using the Prokaryotic Genome Annotation Pipeline (8), and the annotated genome was submitted to GhostKOALA (9) for biochemical pathway analysis.

The draft genome of LSUCC0112 is 3,534,449 bp, consisting of 19 contigs, with an *N*_50_ value of 940,886, and a final coverage of 23.8×. It has a G+C content of 52.5% and is estimated to be 97.04% complete with 1.37% contamination. The genome has a total of 3,141 predicted genes, of which 3,054 are protein coding, 41 tRNAs, 6 rRNA genes (2 copies of the 5S, 16S, and 23S rRNA genes), and 87 predicted pseudogenes. The draft genome encodes both high- and low-affinity cytochrome c oxidases for aerobic respiration (*ccoOPQN* and *coxABC*, respectively), as well as complete glycolytic, pentose-phosphate, and oxidative citric acid cycle pathways. LSUCC0112 shows potential glycogen synthesis (*glgABC*) and response genes for phosphate (*phoABDR*) and nitrogen limitation (*glnADGL*). The draft genome also carries genes for assimilatory sulfate reduction and chemotaxis (*cheABRWVY*), including flagellar assembly.

### Data availability.

Raw fastq sequences have been deposited to the NCBI short read archive under the accession number SRR8923645. The assembly has BioProject number PRJNA528512 and GenBank assembly accession number GCA_004535785.
